# COVID-19 Awareness Among Healthcare Students and Professionals in Mumbai Metropolitan Region: A Questionnaire-Based Survey

**DOI:** 10.7759/cureus.7514

**Published:** 2020-04-02

**Authors:** Pranav D Modi, Girija Nair, Abhay Uppe, Janhavi Modi, Balaji Tuppekar, Amit S Gharpure, Deepak Langade

**Affiliations:** 1 Pulmonary Medicine, D Y Patil Hospital, Navi Mumbai, IND; 2 Pulmonology, D Y Patil Hospital, Navi Mumbai, IND; 3 Oral and Maxilofacial Surgery, Y.M.T. Dental College and Hospital, Navi Mumbai, IND; 4 Periodontics, University of Washington School of Dentistry, Seattle, USA; 5 Pharmacology, D Y Patil University - School of Medicine, Navi Mumbai, IND

**Keywords:** covid-19 india, covid-19 mumbai, coronavirus, health care workers, who coronavirus, cdc coronavirus, covid-19, healthcare professionals, ppe, hand hygiene

## Abstract

Background and objectives

The rapid and extensive spread of the COVID-19 pandemic has become a major cause of concern for the healthcare profession. The aim of this study is to assess the awareness of COVID-19 disease and related infection control practices among healthcare professionals and students in the Mumbai Metropolitan Region.

Materials and methods

A total of 1562 responders from the Mumbai Metropolitan Region completed a questionnaire-based survey on the awareness, knowledge, and infection control practices related to COVID-19 infection in the healthcare setting. The questionnaire was adapted from the current interim guidance and information for healthcare workers published by the US Centers for Disease Control and Prevention (CDC). Convenient sampling method was used for data collection and the distribution of responses was presented as frequencies and percentages. Descriptive statistics were performed for all groups and subgroups based on the percentage of correct responses. Individual pairwise comparisons were done using the median test for the percentage of correct responses.

Results

The overall awareness for all subgroups was adequate with 71.2% reporting correct answers. The highest percentage of correct responses were from undergraduate medical students and the lowest was from non-clinical/administrative staff. Less than half of the total respondents could correctly define “close contact.” More than three-fourths of the responders were aware of the various infection control measures like rapid triage, respiratory hygiene, and cough etiquette and having a separate, well ventilated waiting area for suspected COVID-19 patients. However, only 45.4% of the responders were aware of the correct sequence for the application of a mask/respirator, and only 52.5% of the responders were aware of the preferred hand hygiene method for visibly soiled hands.

Conclusion

There is a need for regular educational interventions and training programs on infection control practices for COVID-19 across all healthcare professions. Occupational health and safety are of paramount importance to minimize the risk of transmission to healthcare students and professionals and provide optimal care for patients.

## Introduction

India braces for the COVID-19 pandemic; healthcare workers on the frontlines are particularly vulnerable to this infection. The virus that causes COVID -19 was initially called as 2019-nCoV and was then termed as syndrome coronavirus 2 (SARS-CoV-2) by the International Committee on Taxonomy of Viruses (ICTV) [1]. It is a new strain discovered in 2019 which was not found previously in humans.

Previously, the severe acute respiratory syndrome-coronavirus (SARS-CoV) and the Middle East respiratory syndrome-coronavirus (MERS-CoV) have been known to affect humans. Outbreaks of respiratory disease caused by these viruses seem to have originated in animals before moving into other hosts like humans. MERS-CoV was found to be transmitted from Arabian camels to humans, whereas SARS-CoV was transmitted from civet cats to humans. SARS-CoV-2 seems to have originated from bats and first reports of cases were from Wuhan, Hubei Province in China, suggesting an animal-to-person spread from a live animal market. The virus then spread outside Hubei and subsequently, to the rest of the world via human transmission. Several countries have now reported community spread. The World Health Organization (WHO) declared coronavirus disease as a pandemic on March 11, 2020 [2].

With this mode of transmission, healthcare workers are among the highest risk of being infected. The highly contagious SARS-CoV-2 virus is an additional hazard for the healthcare system apart from the burden of extended work hours, physical and psychological stress, burnout, and fatigue [3]. The objective of this study is to assess the awareness of COVID-19 disease and its related infection control practices among healthcare professionals in the Indian healthcare scenario. This was a questionnaire-based survey adapted from current interim guidelines and information for healthcare personnel provided by the US Centers for Disease Control and Prevention (CDC) and WHO.

## Materials and methods

This survey was conducted at a tertiary-care hospital and teaching institute in Navi Mumbai. The survey was prepared in the form of an online form and was sent to 4450 potential responders who included students and staff at various healthcare institutions in the Mumbai Metropolitan Region in the state of Maharashtra, India. The period of the survey was March 12-19, 2020, and a total of 1562 responders completed the survey with a response rate of 35.1%.

The self-administered questionnaire consisting of socio-demographic questions, and 17 questions based on knowledge and infection control practices related to COVID-19 disease in the healthcare setting were adapted from the current interim guidance and information for healthcare workers published by the CDC, updated on March 7, 2020 [4]. The questionnaire also included questions related to hand hygiene techniques based on the “five moments of hand hygiene” described by the WHO, which were used to test participants’ knowledge in optimal hygiene practices [5].

Consent was obtained by all participants in this study. The Institutional Ethics Committee (IEC) reviewed and approved the study-related documents (DYP/IECBH/2020/01). Convenient sampling method was used for data collection, and the distribution of responses was presented as frequency and percentages. Sub-groups were classified on the basis of gender, age (18-30 years, 31-45 years, and >45 years) and profession (undergraduate, graduate students and faculty from medical, dental nursing, and physical therapy schools and institutes, non-clinical staff and administrators, paramedical staff, and professionals from the allied health sciences). Sub-groups were also classified on the basis of the training received by the responders for hand hygiene procedures. Data were tabulated in excel, and descriptive statistics were performed using SPSS 17. Individual pairwise comparisons were done using the median test for percent correct response.

## Results

A total of 1562 healthcare professionals from the Mumbai Metropolitan Region responded to the survey. The majority of the responders were from the age group of 18-30 years (n = 1136). Approximately 75.9% (n = 1,185) of the responders were females and 83.5% of the responders were from the city of Navi Mumbai. Among the various sub-groups, 33.1% (n = 517) of the medical students, 24.3% (n =379) of the nursing staff and students and 10.9% (n = 171) of the medical postgraduates, fellows, and faculty completed the survey (Table 1).

**Table 1:** Responder profile

Demographic Group	Sub-Group	No.	%
Age group	18-30 yrs	1376	88.1
	31-45 yrs	120	7.7
	>45 yrs	66	4.2
	Total	1562	100
Gender	Male	377	24.1
	Female	1185	75.9
	Total	1562	100
Location	Mumbai: South & Central	91	5.8
	Mumbai: Western	77	4.9
	Navi Mumbai	1304	83.5
	Others	90	5.8
Total		1562	100
Profession	Allied Health Sciences	141	9
	Dentistry (students and faculty)	142	9.1
	Medical Post-Graduates	172	10.9
	Medical Students	517	33.1
	Non-clinical/admin staff	12	8
	Nursing (students and faculty)	379	24.3
	Paramedical staff	37	2.4
	Physiotherapy/Occupational therapy (students and faculty)	163	10.4
Total		1562	100

The responses of various professional sub-groups, gender, and age have been presented in Tables 2 and 3.

**Table 2:** Percentage of correct responses in different age groups and gender

	Age						Gender					
	18 to 30 years (n = 1376)		31 to 44 years (n = 120)		45 and above (n = 66)		Female (n = 1185)		Male (n = 377)		Total (n = 1562)	
	No.	%	No.	%	No.	%	No.	%	No.	%	No.	%
Q-1	185	13.4%	7	5.8%	6	9.1%	151	12.7%	47	12.5%	198	12.68%
Q-2	1361	98.9%	120	100.0%	63	95.5%	1170	98.7%	374	99.2%	1544	98.85%
Q-3	872	63.4%	70	58.3%	27	40.9%	738	62.3%	231	61.3%	969	62.04%
Q-4	608	44.2%	53	44.2%	31	47.0%	512	43.2%	180	47.7%	692	44.30%
Q-5	1200	87.2%	102	85.0%	58	87.9%	1033	87.2%	327	86.7%	1360	87.07%
Q-6 Yes - Received Prior Hand Hygiene Training	209	15.2%	21	17.5%	28	42.4%	202	17.0%	56	14.9%		
Q-6 No - Not Received Prior Hand Hygiene Training	1167	84.8%	99	82.5%	38	57.6%	983	83.0%	321	85.1%		
Q-7	1211	88.0%	107	89.2%	55	83.3%	1048	88.4%	325	86.2%	1373	87.90%
Q-8	854	62.1%	87	72.5%	44	66.7%	757	63.9%	228	60.5%	985	63.06%
Q-9	1072	77.9%	102	85.0%	52	78.8%	924	78.0%	302	80.1%	1226	78.49%
Q-10	1247	90.6%	115	95.8%	65	98.5%	1074	90.6%	353	93.6%	1427	91.36%
Q-11	1197	87.0%	111	92.5%	64	97.0%	1033	87.2%	339	89.9%	1372	87.84%
Q-12	1092	79.4%	91	75.8%	51	77.3%	930	78.5%	304	80.6%	1234	79.00%
Q-13	751	54.6%	67	55.8%	34	51.5%	630	53.2%	222	58.9%	852	54.55%
Q-14	566	41.1%	46	38.3%	19	28.8%	467	39.4%	164	43.5%	631	40.40%
Q-15	1027	74.6%	100	83.3%	55	83.3%	892	75.3%	290	76.9%	1182	75.67%
Q-16	1240	90.1%	112	93.3%	59	89.4%	1057	89.2%	354	93.9%	1411	90.33%
Q-17	1204	87.5%	100	83.3%	54	81.8%	1026	86.6%	332	88.1%	1358	86.94%
Overall correct percentage (Median)		71.2%		72.4%		69.8%		74.8%		72.5%		71.3%

 

**Table 3:** Distribution of correct responses according to profession

	Profession															
	Allied health sciences (n = 141)		Dentistry (Students and faculty) (n = 142)		Medical Post-graduates (residents, fellows, faculty) (n = 171)		Medical Students (n = 517)		Non-clinical/administrative staff (n = 12)		Nursing (Students & faculty) (n = 379)		Paramedical staff (n = 37)		Physiotherapy, occupational therapy (students and faculty) (n = 163)	
	N	%	N	%	N	%	N	%	N	%	N	%	N	%	N	%
Q-1	20	14.2%	7	4.9%	9	5.3%	55	10.6%	2	16.7%	69	18.2%	12	32.4%	24	14.7%
Q-2	136	96.5%	142	100.0%	168	98.2%	515	99.6%	10	83.3%	374	98.7%	36	97.3%	163	100.0%
Q-3	81	57.4%	77	54.2%	97	56.7%	363	70.2%	2	16.7%	229	60.4%	28	75.7%	92	56.4%
Q-4	54	38.3%	65	45.8%	92	53.8%	253	48.9%	2	16.7%	140	36.9%	14	37.8%	72	44.2%
Q-5	120	85.1%	135	95.1%	151	88.3%	474	91.7%	8	66.7%	294	77.6%	26	70.3%	152	93.3%
Q-6 Yes - Received prior hand hygiene training	28	19.9%	39	27.5%	33	19.3%	78	15.1%	4	33.3%	15	4.0%	0	0.0%	61	37.4%
Q-6 No - Did not receive prior hand hygiene training	113	80.1%	103	72.5%	138	80.7%	439	84.9%	8	66.7%	364	96.0%	37	100.0%	102	62.6%
Q-7	116	82.3%	121	85.2%	155	90.6%	466	90.1%	9	75.0%	336	88.7%	32	86.5%	138	84.7%
Q-8	60	42.6%	108	76.1%	113	66.1%	361	69.8%	4	33.3%	200	52.8%	27	73.0%	112	68.7%
Q-9	96	68.1%	130	91.5%	153	89.5%	429	83.0%	7	58.3%	251	66.2%	26	70.3%	134	82.2%
Q-10	111	78.7%	136	95.8%	169	98.8%	494	95.6%	9	75.0%	320	84.4%	33	89.2%	155	95.1%
Q-11	128	90.8%	129	90.8%	163	95.3%	449	86.8%	9	75.0%	327	86.3%	30	81.1%	137	84.0%
Q-12	114	80.9%	129	90.8%	121	70.8%	403	77.9%	9	75.0%	291	76.8%	27	73.0%	140	85.9%
Q-13	70	49.6%	86	60.6%	78	45.6%	301	58.2%	7	58.3%	200	52.8%	12	32.4%	98	60.1%
Q-14	58	41.1%	50	35.2%	65	38.0%	218	42.2%	2	16.7%	163	43.0%	12	32.4%	63	38.7%
Q-15	96	68.1%	121	85.2%	141	82.5%	415	80.3%	3	25.0%	246	64.9%	25	67.6%	135	82.8%
Q-16	121	85.8%	133	93.7%	164	95.9%	490	94.8%	10	83.3%	314	82.8%	29	78.4%	150	92.0%
Q-17	125	88.7%	112	78.9%	135	78.9%	450	87.0%	10	83.3%	347	91.6%	36	97.3%	143	87.7%
Overall correct percentage (Median)		66.7%		73.9%		72.1%		74.1%		53.6%		67.6%		68.4%		73.1%

Only 22.6% of the responders were aware that the virus causing COVID-19 was initially called as 2019-nCoV and was later termed as syndrome coronavirus 2 (SARS-CoV-2). The main mode of transmission of the virus is via respiratory droplets which were answered correctly by 62% of the responders, with the lowest percentage of correct answers coming from the non-clinical/administrative staff sub-group (16.7%).

Only 48.9% (n = 692) of the total respondents were able to correctly define “close contact”. The highest number of correct responses were from the medical undergraduate student sub-group (48.9%; n = 253), and the lowest number was from the non-clinical/administrative staff sub-group (16.7%). A majority (87.1% ) of the responders were able to correctly answer questions related to COVID-19 exposure that required medical attention.

**Figure 1:**
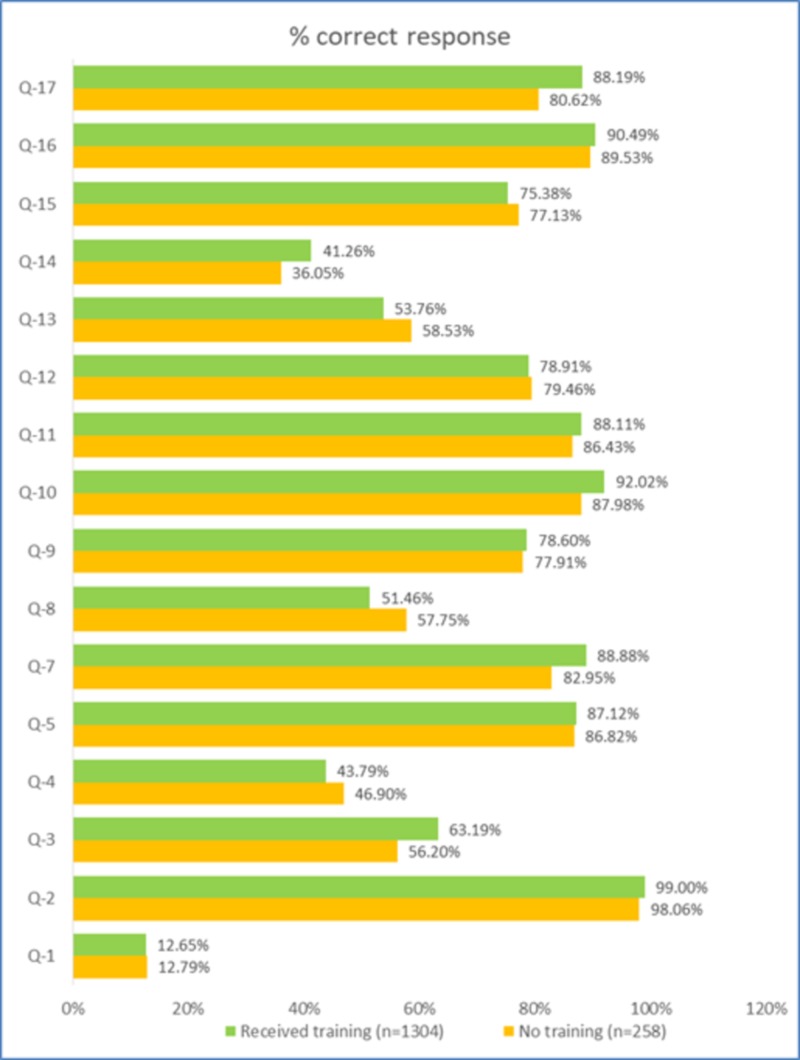
Percent correct responses (median) for all items in those who received training and those who did not (p>0.05 for all pairwise comparisons, median test)

Approximately 83% of the responders had received formal hand hygiene treatment in the last three years and 87.9% were aware of the moments of hand hygiene. However, only 52.5% of the responders were aware of the preferred hand hygiene method for visibly soiled hands. Interestingly, a higher number of correct responses were from the group which did not receive any formal hand hygiene training (Figure 1).

Seventy-nine percent of the responders were aware of the various personal protective equipment (PPE) recommended for use in suspected COVID-19 patients in a healthcare setting. Eighty-seven percent were aware of the essential PPE needed for transporting a suspected patient within a healthcare facility. Only 54.5% of the responders were aware of the isolation procedures necessary for a confirmed COVID-19 patient (Airborne Infection Isolation Room without exhaust). Out of the various sub-groups, the physiotherapy and occupational therapy group had the highest number of correct responses (60.1%), whereas the lowest number was from paramedical staff sub-group (32.4%). Eighty-seven percent of the responders were aware of the recommended infection prevention and control measures to perform aerosol-generating procedures.

Only 45.4% of the responders were aware of the right sequence for the application of a mask/respirator. The highest number of correct responses were from the nursing sub-group (43%) and undergraduate medical student sub-group (42.2%), whereas the lowest number was from the paramedical staff (32.4%) and non-clinical/administrative staff (16.7%) sub-groups.

More than 75% of the responders were aware of the various infection control measures like rapid triage, respiratory hygiene, and cough etiquette and having a separate, well-ventilated waiting area for suspected COVID-19 patients.

**Figure 2:**
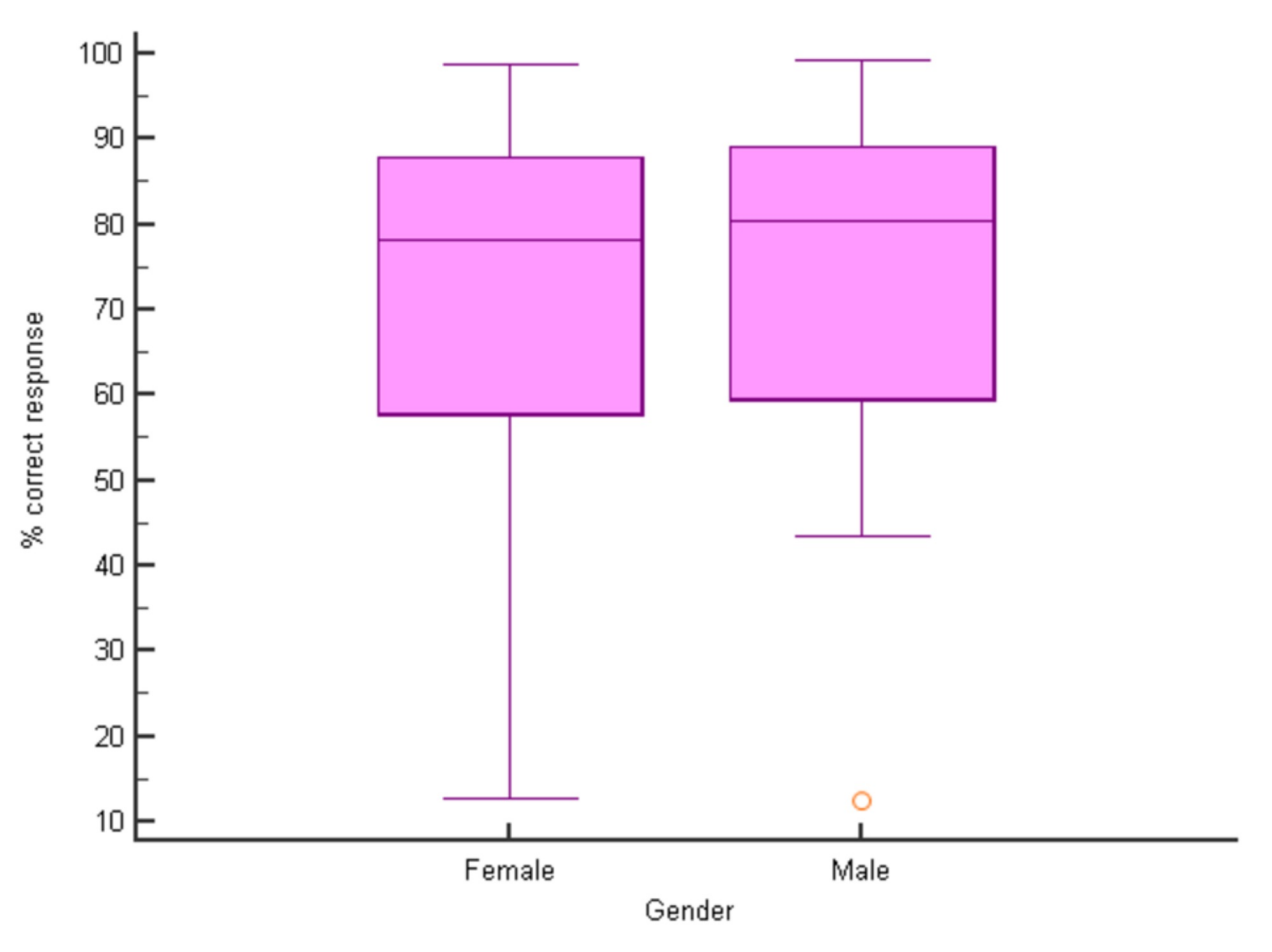
Percent correct responses (median) for males and females (p > 0.05 for all pairwise comparisons, median test)

**Figure 3:**
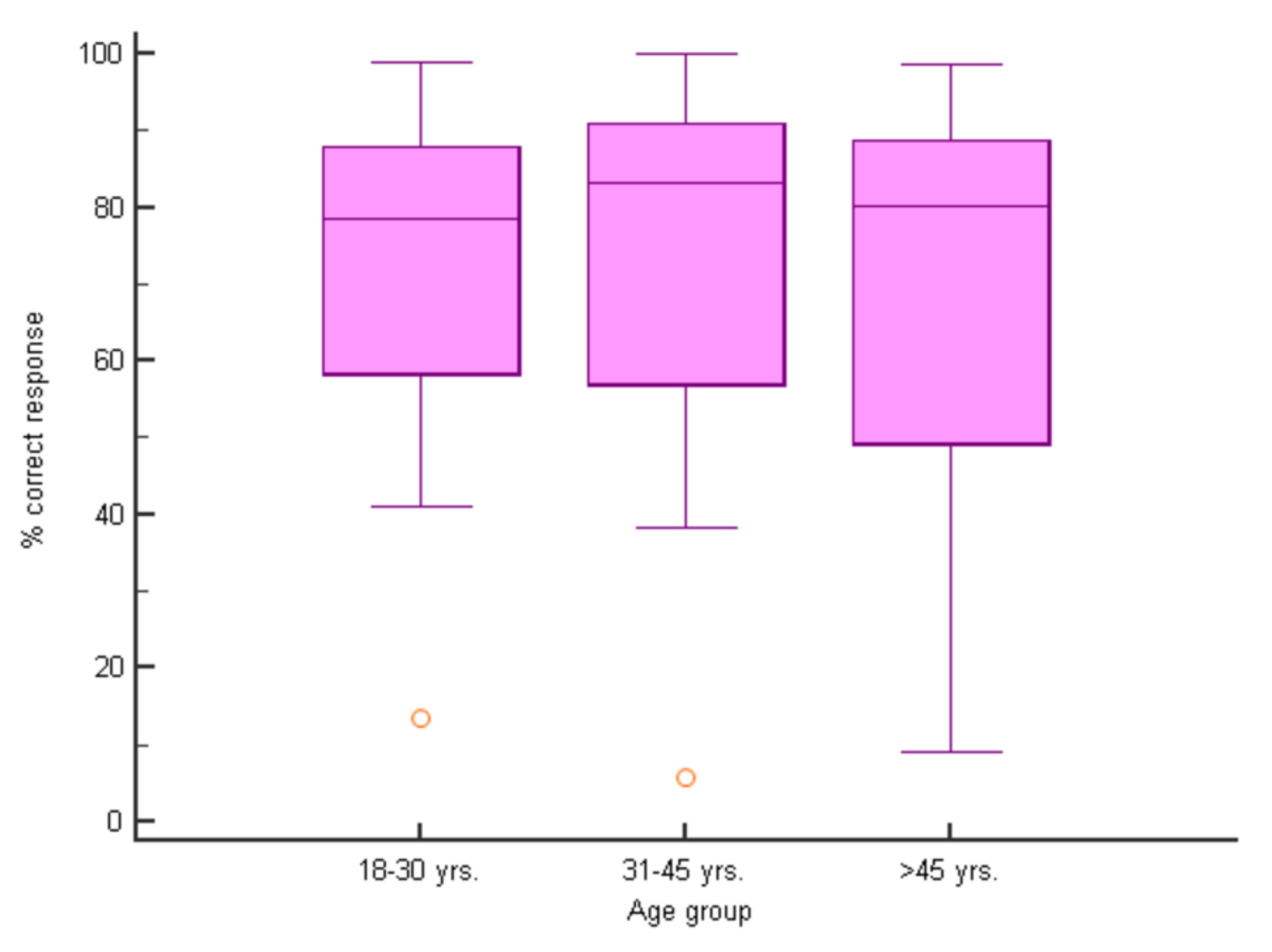
Percent correct responses (median) for different age groups (p>0.05 for all pairwise comparisons, median test)

**Figure 4:**
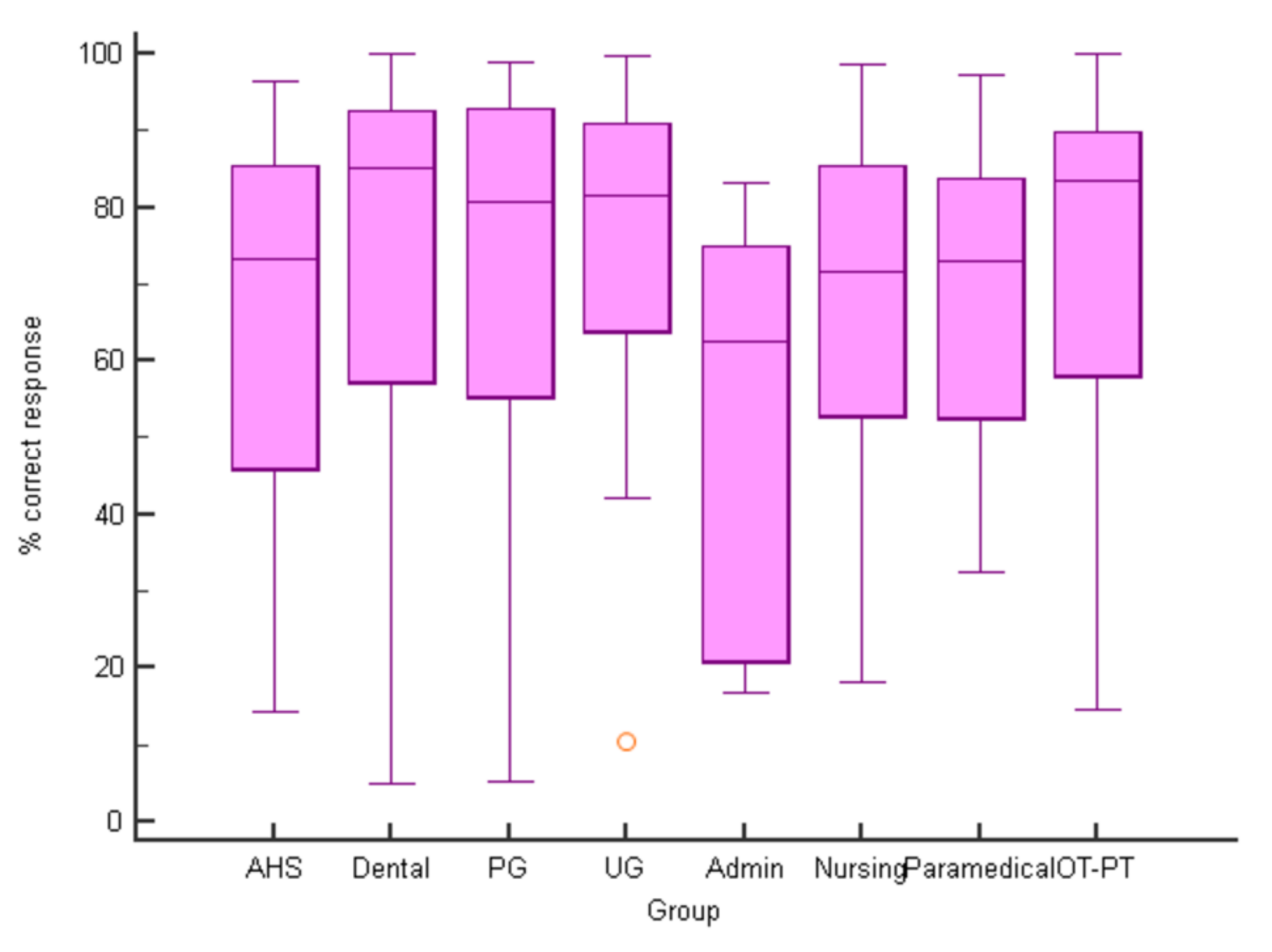
Percent correct responses (median) for different responders (p>0.05 for all pairwise comparisons, median test) AHS, Allied Health Sciences; UG, Undergraduate Medical Students; PG, Post-graduate Medical Students; OT-PT, Occupational and Physical Therapy Students

The overall percentage of correct answers for all groups was 71.2%. A slightly higher percentage of correct responses were from the age sub-group of 18-30 (72.38%) and from females (74.78%). A higher percentage of correct answers were obtained from medical students, dentists, physiotherapy and occupational therapy group sub-groups (Figures 2-4).

## Discussion

Since its initial outbreak in China in December 2019, the COVID-19 disease has had a cascading effect worldwide. According to the ICMR update on March 23, 2020, more than 400 individuals have been confirmed positive in India [6]. The identification and isolation of a suspected case is the most important step in curbing the spread of COVID-19. However, in our study, less than half of the responders were aware of defining a "close contact." According to the US CDC, a "close contact" is defined as: “being within approximately 6 feet (2 meters) of a COVID-19 case for a prolonged period of time or having direct contact with infectious secretions of a COVID-19 case. Similarly, various other key definitions have been provided in Interim U.S. Guidance for Risk Assessment and Public Health Management of Healthcare Personnel with Potential Exposure in a Healthcare Setting to Patients with Coronavirus Disease (COVID-19) published by the CDC [4]. Awareness was low among all subgroups with the lowest being the non-clinical/administrative staff. Even though this group is not actively involved in patient management, there are high chances of non-clinical staff having patient contact at some point in the healthcare setting and therefore at risk of contracting and spreading the infection.

Correct hand hygiene practices play a crucial role in preventing the spread of infection. The WHO “Five Moments of hand hygiene” defines key moments when healthcare providers must carry out hand hygiene [7]. Two basic methods to clean hands are hand washing and hand rubbing. The CDC recommends alcohol-based hand rub (ABHR) in most situations [8]. However, the question in our survey was focussed on the recommended hand hygiene technique for visibly soiled hands which is handwashing with soap and water for at least 20 seconds with the whole process lasting for up to 40-60 seconds [5].

Awareness of the use of personal protective equipment (PPE) for suspected/confirmed COVID-19 cases was high among all groups of healthcare professionals. The CDC has provided Interim Infection Prevention and Control Recommendations for Patients with suspected or confirmed coronavirus disease 2019 (COVID-19) in Healthcare Settings for PPE [9]. A Facemask/N95 respirator should be used when entering into the patient room. The N95 respirator is preferred over face mask when performing or presents for aerosol-generating procedures. Proper disposal of the used masks and hand hygiene should be performed. A clean gown with goggles or disposable face shield and clean non- sterile gloves are recommended upon entry to the patient room area. In case of shortage, gowns should be prioritized for aerosol-generating procedures.

**Figure 5:**
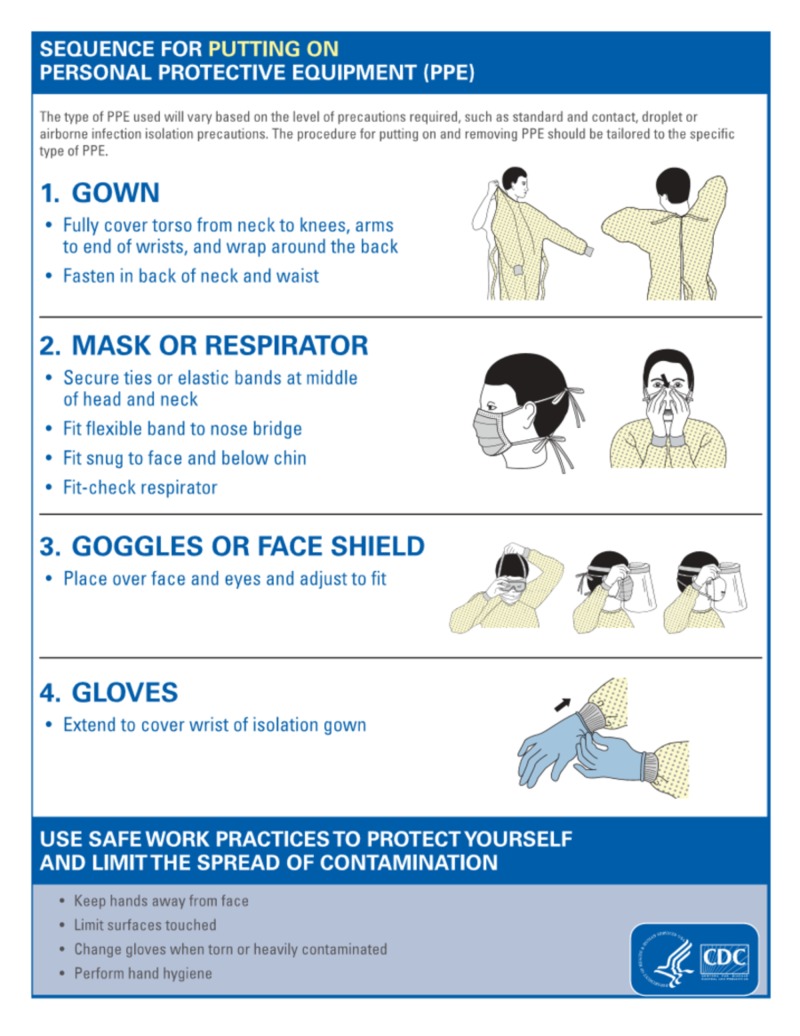
Sequence for putting on PPE This material was developed by The Centers for Disease Control and Prevention (https://www.cdc.gov/hai/pdfs/ppe/ppe-sequence.pdf) PPE, personal protective equipment

Besides being aware of the required PPE, it is also important to know the correct sequence of “donning and doffing” of PPE. The CDC sequence of donning a face mask is as follows: securing ties or elastic bands at the middle of head and neck, fitting the flexible band to the nose bridge, fit snug to face and below the chin, fit-check respirator [10] (Figure 5).

More than 75% of the respondents were of the opinion that the use of a facemask/respirator is not essential or recommended for people who are well and not in contact with a suspected or infected COVID-19 patient. The general recommendation from all major global health organizations is that those in healthcare settings or those who are symptomatic should use a mask. Even though discrepancies have been observed for use in the community setting, the widespread use of masks should be discouraged to preserve limited supplies for healthcare settings [11].

Patient isolation and aerosol performing procedures should be carried out in the Airborne Infection Isolation Room (AIIR). These are rooms kept under negative pressure. Suspected or confirmed patients should not be placed in a room that has an exhaust that recirculates air within the hospital building. Air from these rooms should be filtered through a high-efficiency particulate air (HEPA) filter directly before recirculation. Less than half the responders in our survey were aware of this concept.

The overall percentage of correct answers for our study participants was 71.2% with the highest percentage of correct responses from medical undergraduate students (74.10%) and lowest from the non-clinical/administrative staff (53.64%). A cross-sectional study regarding knowledge and attitudes towards Middle East respiratory syndrome-coronavirus (MERS-CoV) was conducted on healthcare workers in primary healthcare centers and hospitals at Najran in Saudi Arabia which showed a majority of the healthcare workers were aware of MERS-CoV and had sufficient knowledge regarding the same. Physicians and nurses had significantly better knowledge compared with other healthcare workers [12]. The results of a similar survey carried out in healthcare workers in the Kingdom of Saudi Arabia suggested poor knowledge about emerging infectious diseases among study participants, and self-reported infection control practices were found to be sub-optimal. In South Korea, a survey study of healthcare workers suggested a poor level of knowledge of the modes of transmission of MERS coronavirus [13].

To the best of our knowledge, this is the first study that evaluates the awareness of COVID-19 among Indian healthcare students and professionals. In the midst of this crisis, the Indian health ministry has proposed to provisionally permit medical undergraduates of senior grades to treat COVID-19 patients [14]. This move could help plug the shortage of healthcare professionals and potentially provide care to a large number of people. Hence, students from various healthcare professions were included in our study.

The current situation demands urgent development of strategies to prevent infection among high-risk populations including pre-exposure and post-exposure prophylaxis. Various drugs including antivirals and antimalarials are under trial currently. In vitro drug testing has shown the antimalarial hydroxychloroquine to have antiviral activity against SARS-CoV-2 and could be potentially used as chemoprophylaxis for healthcare workers. Clinical trials for the treatment of COVID-19 pneumonia with hydroxychloroquine are underway and results of the same will be monitored closely in the coming days [15].

One of the drawbacks of this study is that most respondents are from urban locations in the Mumbai Metropolitan Region which do not truly represent the healthcare professionals of the entire state and country.

## Conclusions

Healthcare professionals and students from the Mumbai Metropolitan Region showed adequate awareness of COVID-19 in the healthcare setting with an overall percentage of 71.2% correct answers. A higher percentage of correct responses were from undergraduate medical students and the lowest was from non-clinical/administrative staff. This study shows that there is a strong need to implement periodic educational interventions and training programs on infection control practices for COVID-19 across all healthcare professions. Conducting periodic webinars for educational intervention for all healthcare students and professionals including non-clinical and administrative staff, paramedical and nursing sub-groups could be a useful and safe tool to create more awareness.

Disclaimer: This article was last updated on March 23, 2020, and it may not be updated regularly. COVID-19 is an emerging, rapidly evolving situation and we recommend healthcare students and professionals to review the latest official information from local governments and health organizations.

## Figures and Tables

**Figure 6:**
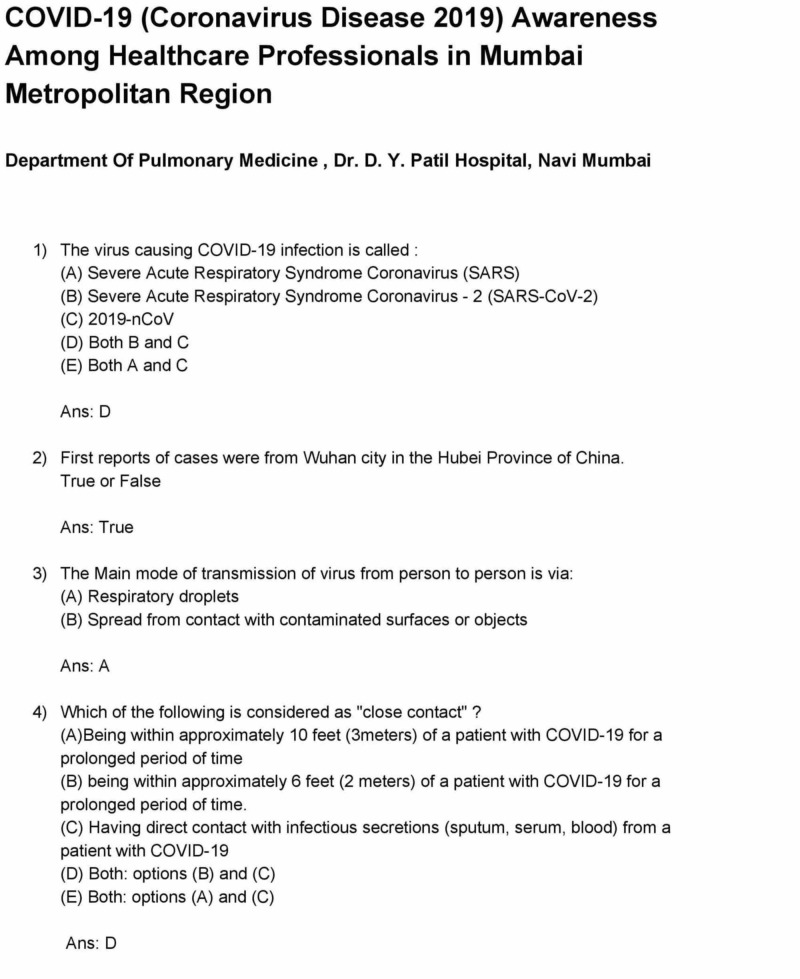
Questionnaire page 1

**Figure 7:**
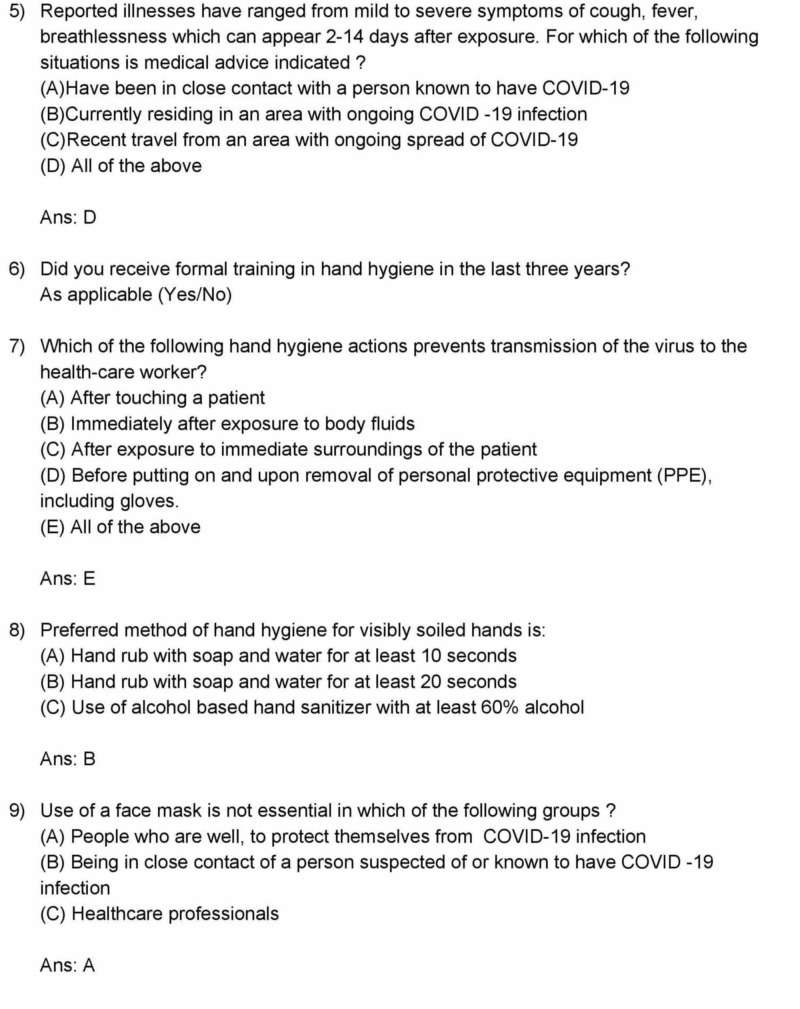
Questionnaire page 2

**Figure 8:**
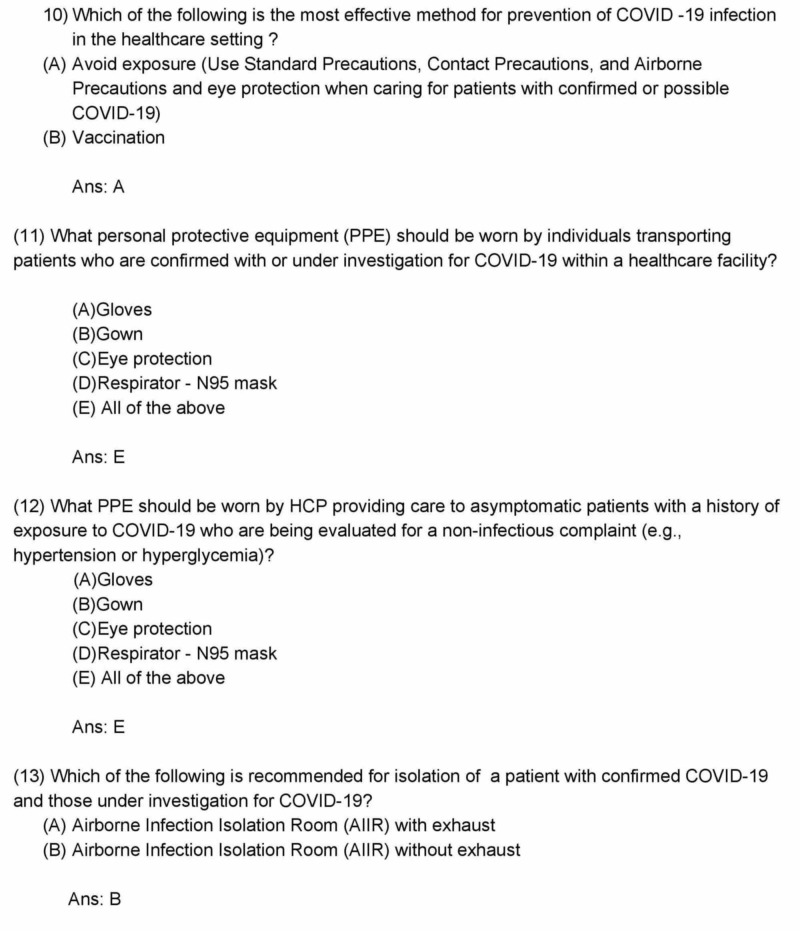
Questionnaire page 3

**Figure 9:**
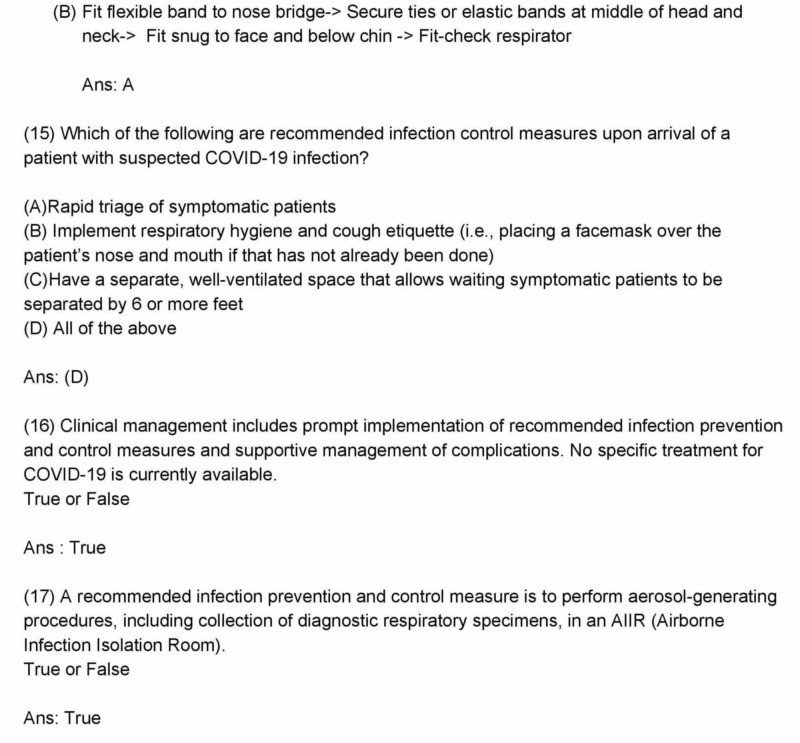
Questionnaire page 4

## Appendices

Supplementary material: COVID-19 Awareness in Healthcare Professionals - Questionnaire
